# Irregularly Irregular Wide Complex Tachycardia: Look Beyond the Atrium

**DOI:** 10.1016/j.jaccas.2026.108755

**Published:** 2026-06-23

**Authors:** Gaurav Lakhani, Daljeet Kaur Saggu, Muthiah Subramanian, Sachin Yalagudri, Calambur Narasimhan

**Affiliations:** AIG Hospitals, Hyderabad, India

**Keywords:** atrial fibrillation, electrocardiogram, electrophysiology, ventricular tachycardia

## Abstract

**Case Summary:**

A 69-year-old gentleman, a patient with ischemic cardiomyopathy with a single-chamber implantable cardioverter-defibrillator, presented with palpitations. Electrocardiogram revealed a wide QRS, irregularly irregular rhythm (>50 ms variability), mimicking atrial fibrillation but favoring ventricular tachycardia (VT) via the Basel algorithm, superior axis, morphology criteria, and evidence of atrioventricular dissociation. Electrophysiology study confirmed inducible irregular VTs; successful substrate-based catheter ablation was performed.

**Take-Home Messages:**

Irregularly irregular wide complex tachycardia in structural heart disease warrants consideration of VT despite irregularity, using integrated algorithms like the Basel algorithm and morphology criteria. In patients with documented irregularly irregular VT, it is prudent to disable the stability criterion and rely on onset and morphology criteria for supraventricular tachycardia discrimination in a single-chamber implantable cardioverter-defibrillator.

## Graphical Abstract

**Figure:**
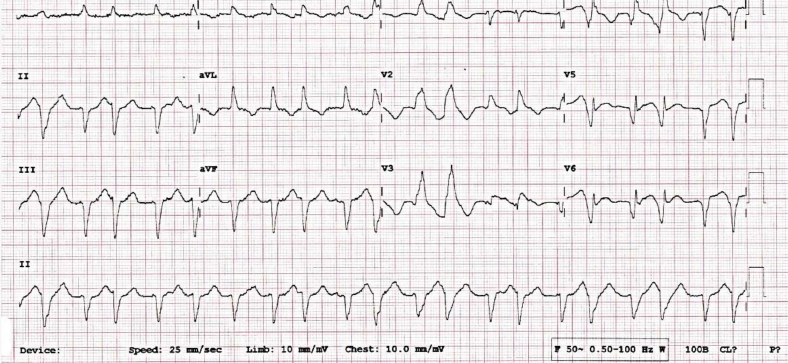

## Background History

A 69-year-old gentleman presented to our clinic with a history of palpitations. His electrocardiogram (ECG) showed a wide QRS, irregularly irregular rhythm with changing QRS morphology (Figure 1) at a rate of 100 to 150 beats/min. The patient was diabetic, hypertensive, and had a history of anterior wall myocardial infarction, for which he underwent multivessel percutaneous coronary intervention followed by single-chamber intracardiac defibrillator implantation (2020) for primary prevention. Two-dimensional echocardiography showed a left ventricular ejection fraction of 30%. Baseline investigations were normal, and he was on guideline-directed medical therapy, including beta-blockers. As per the ECG what is the diagnosis?

1. Atrial fibrillation (AF)
2. Atrial flutter or atrial tachycardia with variable conduction (either aberrancy or antegrade conduction via accessory pathway)
3. Pleomorphic ventricular tachycardia (VT)

**Figure 1:**
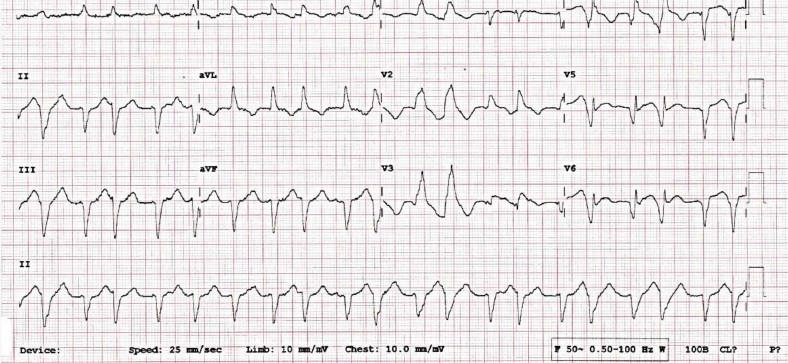
12-Lead Electrocardiogram of the Patient Showing FBIT FBIT = fast broad irregular tachycardia

## Discussion

The ECG shows wide complex tachycardia (WCT) with

1. Irregularly irregular rhythm (cycle length ranging from 400-600 ms; >50 ms variability)
2. Variable bundle branch block (BBB) morphology (lead V1) with superior axis
3. Qr or qS complexes in lead V5, V6
4. Monophasic “R” in V1 with right BBB morphology
5. Sinus “P” wave seen in the ninth beat in rhythm strip
6. >40 ms time to first peak in lead II and avR
7. No distinct P waves in most of the complexes with indirect evidence of atrioventricular dissociation

Except for irregularity of cycle length, all other criteria favor VT in this case (Supplementary Figures 1 and 2). The most common WCT in patients with structural heart disease is VT, which is classically regular and monomorphic. However, irregularity will make us first think of AF with a coexistent conduction abnormality, which can be an underlying BBB, functional BBB, or preexcitation over an accessory pathway. Reverse conduction is the main electrophysiological basis in VT—first through ventricular myocytes and then through the His-Purkinje system, which is sufficient to identify VT, irrespective of whether Vereckei, Brugada, Basel, or any other criteria are used. Different leads merely display different morphological expressions of the same principle. The frontal plane best detects the electrophysiological properties of VT, whereas the horizontal plane confirms and localizes it.

The novel Basel algorithm^1^ is based on both clinical and ECG criteria, which allows rapid and accurate differential diagnosis of WCT (mean 36 seconds vs 105 seconds for the Brugada algorithm and 50 seconds for the Vereckei algorithm). In this algorithm, the presence of structural heart disease as a conduction substrate is an important part, and it defines this as the pretest probability of VT. It takes into account the “time to first peak” measurement in 2 leads, namely II and aVR, which are oriented in nearly opposite directions within the frontal plane, surveying a wide field of activation. This algorithm exhibited similar diagnostic accuracy compared with the Brugada algorithm but with superiority as compared with the Vereckei algorithm (based on single lead aVR). Initial activation of ventricular myocardium followed by depolarization of the His-Purkinje system is an important consideration for the diagnosis of VT. Thus, morphology and other criteria should be taken into consideration before ruling out VT in such cases.

These irregular wide complexes amidst normally conducted QRS complexes should not be mistaken for Ashman's phenomenon.^2^ In patients with AF, aberrant conduction is occasionally seen after a long cycle because of the time dependence of refractoriness on the preceding cycle length and often manifests as right BBB, as the right bundle has a longer refractory period; however, in this case, it is independent of the preceding cycle length.

Our patient underwent an electrophysiology study and catheter ablation. During the electrophysiology study, multiple morphological irregularly irregular VTs (pleomorphic VT) were easily and repeatedly induced. Figures 2A and 2B show intracardiac and 12-lead surface ECGs that are irregularly irregular, changing morphology VTs with atrioventricular dissociation. In view of multiple morphology VTs induced, the scar was defined, and substrate-based ablation was performed (Figure 2C). Post ablation, no VT was induced with programmed extrastimulation from the left ventricle without isoproterenol facilitation (in view of severe left ventricular dysfunction). The patient has now completed 6 months of follow-up without recurrence of VT. Diffuse fibrosis as a part of maladaptive cardiac remodeling may contribute to pleomorphic VT (more than one VT morphology) in any scar-related VT.^3^

**Figure 2:**
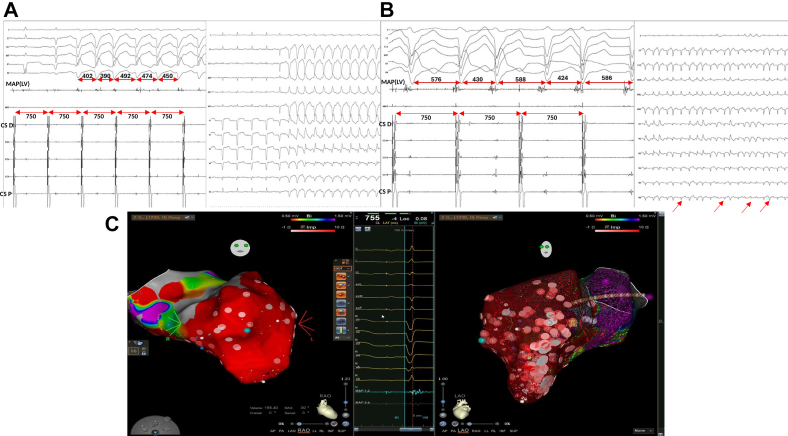
Electrophysiology Study of the Patient (A and B) Shown are intracardiac EGM and 12-lead surface electrocardiograms of irregularly irregular changing morphology (red arrows) VTs induced in the electrophysiology lab with evidence of atrioventricular dissociation. Note: Atrial pacing rate is 750 ms and QRS is changing beat to beat during tachycardia. (C) Three-dimensional CARTO-guided voltage map of left ventricle in right anterior oblique and LAO views with center part of the image showing long fractionated signal (blue EGM) within the scar. Note: The whole anterior wall, apex, and septum are scarred with bipolar voltage <0.5 mV. Substrate-based ablation was performed (red dots in LAO image) within the scar with abnormal signals capturing especially with long stimulus to QRS. EGM = electrogram; LAO = left anterior oblique; VT = ventricular tachycardia.

Diagnostic algorithms of WCT do not take into consideration VT as a differential diagnosis of fast broad irregular tachycardia. Obvious RR variability of more than 50 ms is often the initial step to rule out VT in device detection. In this situation, therapy for VT may be inappropriately withheld due to the stability criteria being turned on, with adverse clinical outcome.^4^ In patients with a single-chamber implantable cardioverter-defibrillator, it would be prudent to avoid the stability criterion to differentiate VT from supraventricular tachycardia. Thus, onset and morphology criteria can be used in cases in which there is documented irregularly irregular VT.

## Funding Support and Author Disclosures

The authors have reported that they have no relationships relevant to the contents of this paper to disclose.
