# Supplementary figures and images for: Irregularly Irregular Wide Complex Tachycardia: Look Beyond the Atrium
Source: JACC Case Rep. 2026 Jun 23;31(30):108755. doi: 10.1016/j.jaccas.2026.108755 (PMC13420549; doi:10.1016/j.jaccas.2026.108755)

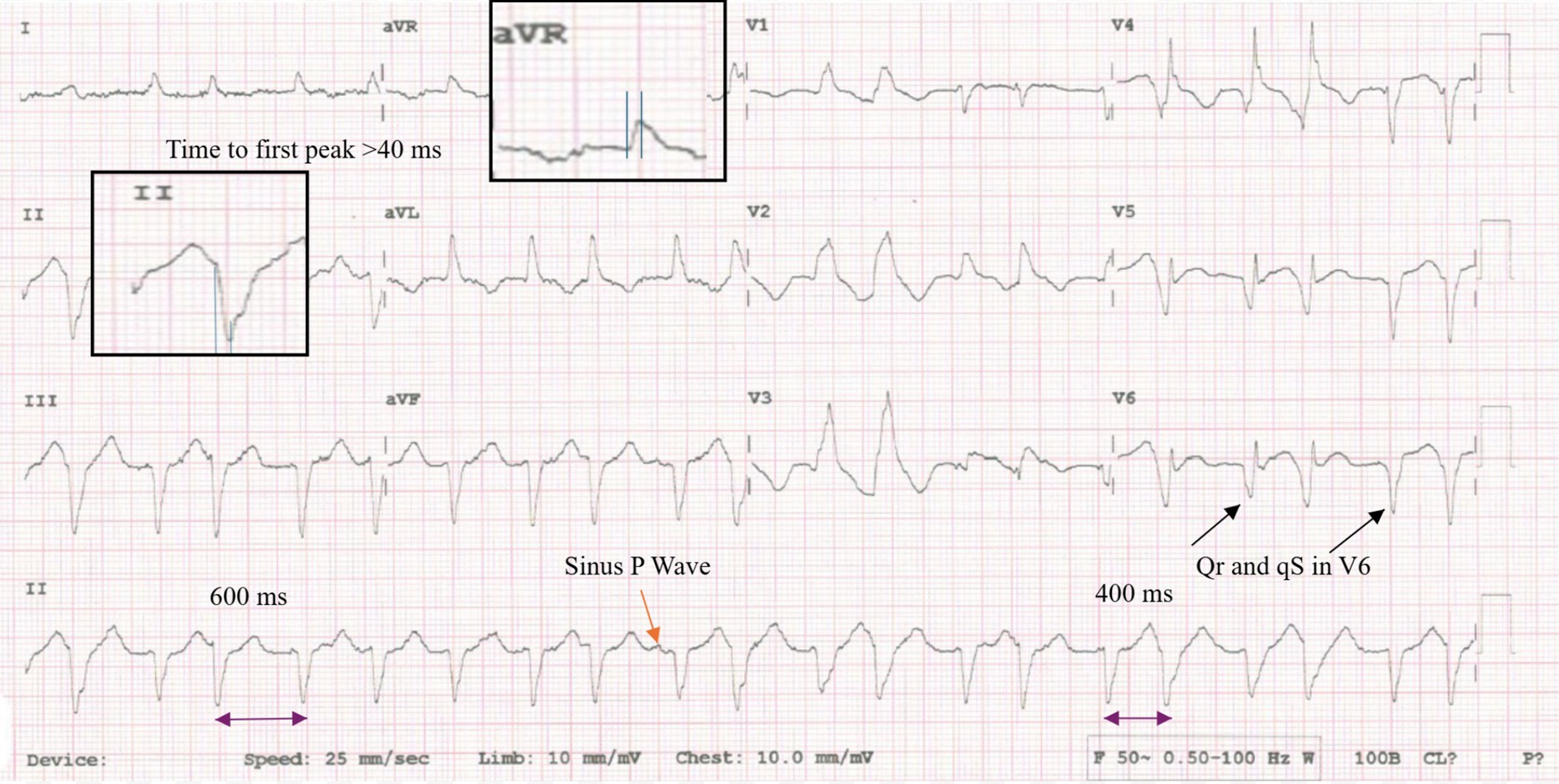

Supplement: Supplemental Figure 1 [file figs1.jpg]

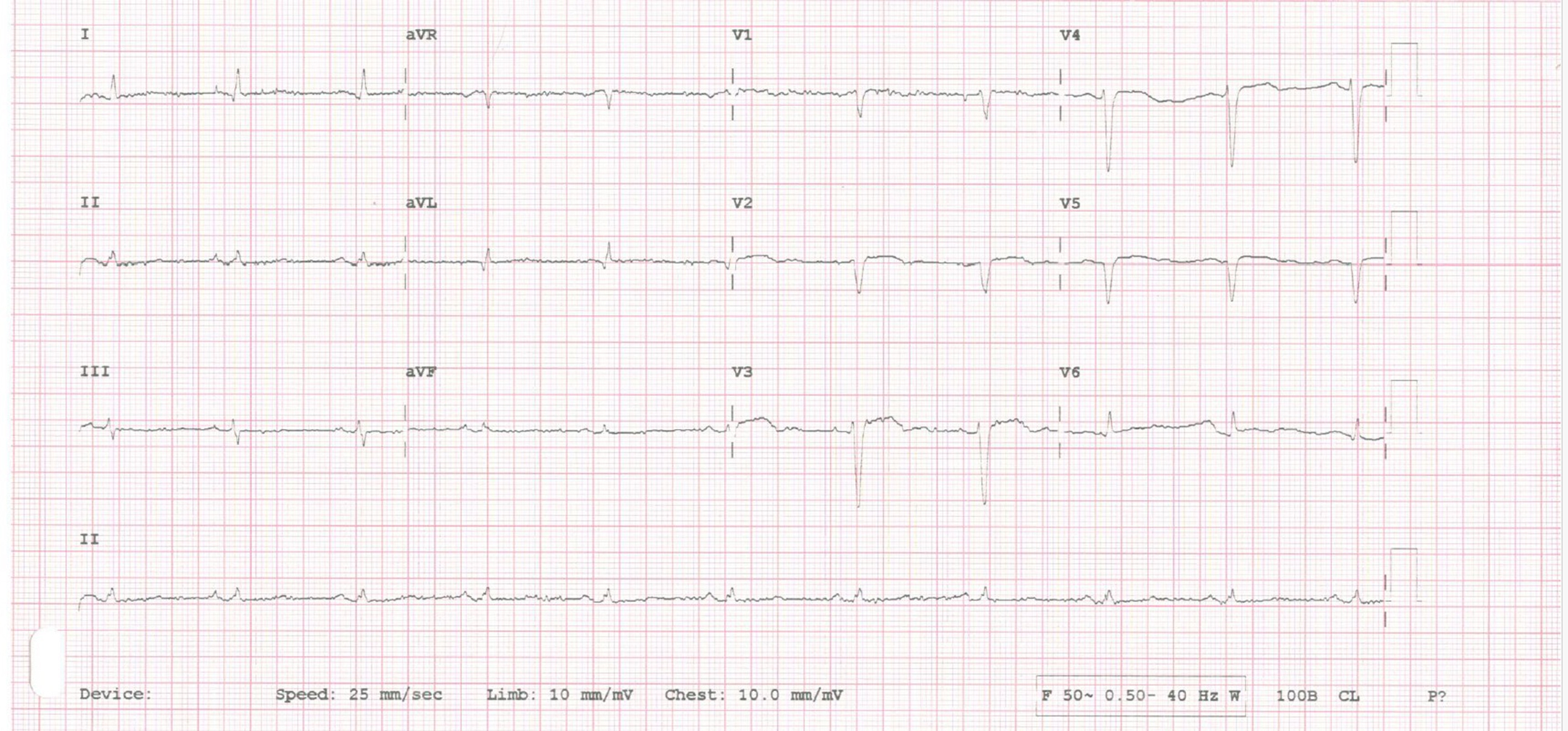

Supplement: Supplemental Figure 2 [file figs2.jpg]
